# The Contribution of Personality and Intelligence Toward Cognitive Competences in Higher Education

**DOI:** 10.3389/fpsyg.2021.621990

**Published:** 2021-07-02

**Authors:** Tania Cerni, Annalisa Di Benedetto, Raffaella I. Rumiati

**Affiliations:** ^1^Dipartimento di Psicologia e Scienze Cognitive, Università di Trento, Rovereto, Italy; ^2^Dipartimento di Scienze Umane, Università degli Studi dell'Aquila, L'Aquila, Italy; ^3^Neuroscience Area, Scuola Internazionale Superiore di Studi Avanzati, Trieste, Italy; ^4^Scuola Superiore di Studi Avanzati Sapienza (SSAS), Sapienza Università di Roma, Roma, Italy

**Keywords:** cognitive competences, personality traits, competence assessment, literacy, numeracy, intelligence, higher education

## Abstract

Personality and cognition are found to be two interrelated concepts and to both have a predictive power on educational and life outcomes. With this study we aimed at evaluating the extent to which personality traits interact with cognition in acquiring cognitive competences during higher education. In a sample of university students at different stages of their career and from different fields of study, we collected Big Five traits, as a measure of personality, and Intelligent Quotient (IQ), as a proxy of cognition. A set of multiple regressions served to explore the relative contribution of IQ and personality traits on the performance on two cognitive competences tests: literacy and numeracy. Results showed that IQ highly modulated numeracy but had a moderate or no impact on literacy while, compared with IQ, personality affects literacy more. In a further explorative analysis, we observed that both the effects of personality and IQ on cognitive competences were modulated by the level of the students' career (freshmen, undergraduates, and bachelor graduates). Different traits, and particularly conscientiousness, increased or decreased their impact on achieved scores depending on the educational level, while IQ lost its effect in undergraduates suggesting that personal dispositions become more influential in advancing the academic carrier. Finally, the field of study resulted to be a predictor of numeracy, but also an important covariate altering the pattern of personality impact.

## Introduction

The concept of competences is becoming central in disciplinary fields related to human and educational development, such as in psychology, pedagogy, sociology, and economics (see Benadusi and Molina, 2018 for a review). This growing attention is driven by the need for appropriate educational and economic policies devoted to potentiating the education system in facing the modern working world. To reach this aim, individual countries, but also wider areas such as the European Union and the Organization for Economic Co-operation and Development (OECD), have been working in developing a complete framework of the fundamental competences as well as assessment programs to evaluate their actual level^1^. Within this framework, competences are conceived in a broad perspective as the ability “to meet complex demands, by drawing on and mobilizing psychosocial resources (including skills and attitudes) in a particular context” (Rychen and Salganik, 2003). Besides *domain-specific* competences (i.e., linked to a disciplinary path or professional profile), particularly relevant are *generic* or *cross-curricular* competences (also referred in the literature as transversal competences), which are common to all disciplinary paths or professional profiles. They include instrumental, interpersonal, and systemic skills and abilities (e.g., González and Wagenaar, 2003; Rychen and Salganik, 2003; Kallioinen, 2010; Rico et al., 2013; Kautz et al., 2014; Hernandez-Linares et al., 2015).

In this work we focused on *cognitive competences*, which represent a subcomponent of those *instrumental* skills and abilities that—together with the interpersonal and systemic aspects—constitute the overall generic competence (González and Wagenaar, 2003; Rychen and Salganik, 2003; Kallioinen, 2010). With respect to the OECD framework, this set of competences corresponds to the “basic” skills such as literacy and numeracy, critical for the development of other competences (OECD, 2012, 2016, but see also chapters 13 and 15 in UNESCO, 2016). Indeed, they are generally described as the ability to understand, evaluate, use and elaborate written text and numerical information in order to face new problems, to achieve personal goals and to develop knowledge. The epithet “cognitive” is tied to the underpinned cognitive constructs that these competences put in place such as problem solving, analytic reasoning, critical thinking (OECD, 2012, 2016). Within the same framework, cognitive competences are usually assessed through specifically designed competence assessment tests, which have been developed with the aim to measure learning outcomes and improve teaching programs. For example, the OECD developed the *Program for International Student Assessment*^2^ (PISA), one of the most famous tests for high schools, and the *Program for the International Assessment of Adult Competences*^3^ (PIAAC), dedicated to adults. These tests are generally designed for target ages, independently of educational curricula, and widely used to compare performance of pupils from different institutions or different countries, meeting the demand for accountability (Rumiati et al., 2018).

Recently, research focusing on the predictive power of cognitive assessments on education and life outcomes (Cappellari et al., 2017), is taking an interest on the skills that are at the core of the measured competences and, in turn, predicted the outcomes on cognitive assessments (e.g., Heckman and Kautz, 2014; Borghans et al., 2016; Jokela et al., 2017). Given the cognitive nature of literacy and numeracy skills, intelligence, as a proxy of cognition, was probably the most investigated at the point that often achievement scores was considered a measure of intelligence (e.g., Murnane et al., 1995; Hanushek and Woessmann, 2008). Among cognitive measures, a high Intelligence Quotient (IQ)—a widely accepted measure of fluid intelligence—was found to contribute to solving newly encountered literacy and numeracy problems independently of formal education (Duckworth et al., 2012; Borghans et al., 2016). However, despite the determinant role of intelligence on cognitive competences, it showed to not exhaustively explain the scores on cognitive assessments. Recent findings showed an important contribution of non-cognitive skills, such as character skills (Heckman and Kautz, 2014). Indeed, not only cognition, but also personal dispositions contribute to cognitive competences. In this context, personality has increasingly become a subject of a stimulating debate. Indeed, advancing that personality plays a key role in determining individual differences in behavior, emotion, motivation but also in cognition (Ackerman, 1996; Allen and DeYoung, 2017), recent research effort has been devoted to evaluating its predictive power on cognitive competence test in order to unveil the contribution of personality traits toward cognitive scores and consequently to educational and life outcomes that these scores predict (Borghans et al., 2011; Salkever, 2015; Lechner et al., 2017). To this end, Borghans et al. (2016) analyzed IQ, school grades, and competence test scores as measures of cognition, and personality as measures of character, all taken from four different datasets. The authors observed that, across datasets, personality better predicts grades and competence test scores, while IQ is better at predicting only the latter, even though most of the variance of both measures is not explained. Moreover, personality predicts grades and scores of competence tests above and beyond IQ on a variety of important life outcomes. Similar results were obtained in a large study with 9-grade students (Lechner et al., 2017). Previous studies reported correlations between IQ and personality (e.g., Duckworth et al., 2011; Rammstedt et al., 2018), grades and IQ (e.g., Ackerman and Heggestad, 1997), personality and grades (Poropat, 2009, 2014), as well as between competence test scores and both personality and IQ (e.g., Duckworth and Seligman, 2005; Duckworth et al., 2012). Overall, these studies suggest that scores on cognitive competence tests are influenced by intelligence as well as personality having proved to be a combined measure of cognitive and non-cognitive skills. In other words, not only cognitive skills are essential in shaping cognitive competences, but also character dispositions drive the acquisition of such competences. According to this view, cognitive assessment tests might tap not only cognitive skills, but also personal, social, and learning skills (European commission, 2006, 2018).

## The Present Study

Despite this interdependence between personality and cognition (Ackerman and Heggestad, 1997; Chamorro-Premuzic and Furnham, 2006), the two constructs were not always considered together as predictors of cognitive assessment scores, but often as predictors of one another (e.g., Duckworth et al., 2011; Soubelet and Salthouse, 2011). Nevertheless, recent findings suggest that these scores are “not pure indicators of cognitive ability or intelligence” (Lechner et al., 2017). As to the role of competence assessment in education, the influence of personality and IQ on cognitive scores has already been investigated in children (e.g., Duckworth et al., 2012) and in adolescents (e.g., Duckworth and Seligman, 2005; Lechner et al., 2017). Given that cognitive assessment tests have become increasingly more common in evaluating students and academic institutions (see Heckman et al., 2014), it is of great interest to know whether and to what extent personality and IQ interact in predicting cognitive competences in higher education. Concerning higher education, the influence of personality on competence assessment scores in the transition between high school to college (Parker et al., 2004), as well as on the academic performance in general (e.g., Chamorro-Premuzic and Furnham, 2003a,b) has received some attention. However, research on the influence of both personality and intelligence on competence test scores during higher education is still lacking. Importantly, while intelligence can be trained with interventions although it showed to be less malleable with age (e.g., Kautz et al., 2014; Protzko, 2017), non-cognitive skills—and generic competences—proved to be malleable in university majors (e.g., Becket and Brookes, 2008; Gallifa and Garriga, 2010; Kouwenhoven, 2010; Pérez Martínez et al., 2010; Hernandez-Linares et al., 2015, 2017; Knipprath, 2017; López et al., 2019). Showing the predictive power of personality traits on cognitive assessment scores during university and in different fields of study might reveal that targeting individual differences, beyond intelligence, in such interventions could indirectly improve cognitive competences.

With the aim to contribute to this issue, with the present study we explored how both personality and intelligence interact in explaining cognitive competences in higher education. As this relationship was previously found at lower educational levels (Duckworth and Seligman, 2005; Duckworth et al., 2012), we hypothesized that both personality and intelligence have a key role in predicting cognitive scores during the academic path and among different fields of study.

In order to pursue our aim, we capitalized on a widely accepted conceptualization of personality traits: the Big Five personality traits or Five-Factor Model: Extraversion, Neuroticism, Agreeableness, Conscientiousness, and Openness/Intellect, commonly measured with self-evaluation questionnaires (Chmielewski and Morgan, 2013; for original conceptualization see e.g., Goldberg, 1990; Costa and McCrae, 1992; John et al., 2008).

The study served several concomitant purposes. First, we aimed at evaluating the relative contribution of IQ, as a proxy of cognition, and Big Five traits, as a measure of personality, on scores collected in a recently designed Italian test devoted to measure literacy and numeracy cognitive competences, the TECO-T. This test belongs to the TECO (TEst of COmpetences) project initiated by the National Agency for the Evaluation of Universities and Research Institutes (ANVUR^4^) in 2013. The aim of the ANVUR was to generate direct indicators reflecting the students' learning outcomes, within the ongoing quality assurance system that includes self-assessment, periodic evaluation, and accreditation of study programs and universities. After several evaluations (see Ciolfi et al., 2016; Damiani et al., 2016, 2017), ANVUR developed and tested the TECO-T—where T stands for *trasversale*, the Italian word for “generic” —, which aim at evaluating generic competences (separated from the TECO-D, with D standing for *disciplinare*, as directly measuring disciplinary competences; Rumiati et al., 2018; Asquini et al., 2019)^5^. We chose the literacy and numeracy TECO-T as it was built to assess exactly what we intended to test: cognitive competences, intended as a subset of generic competences (Rychen and Salganik, 2003). Regarding to our prediction, consistently with the previous research, we expected that competence test scores would be strongly predicted by IQ (Duckworth et al., 2012; Borghans et al., 2016; Lechner et al., 2017). Additionally, given that personality accounts overall for about 5–10% of the variance on cognitive abilities using IQ as a proxy (see Furnham et al., 2007; Rammstedt et al., 2016), we hypothesized to find a similar contribution of the Big Five, in general. In particular, we attempt to confirm the weight of different personality traits on cognitive competences, posing that cognitive measure usually positively correlates with Openness and negatively with Neuroticism and Conscientiousness (see e.g., Ackerman and Heggestad, 1997; Moutafi et al., 2003; Furnham et al., 2007; DeYoung, 2011; Graham and Lachman, 2012;Von Stumm and Ackerman, 2013; Rammstedt et al., 2016; but see also Lounsbury et al., 2005; Baker and Bichsel, 2006; Luciano et al., 2006 for positive correlation with Conscientiousness).

Another explanatory purpose of the study was to test whether the influence of personality and IQ on cognitive scores was modeled by the field of study and the year of education attended. Our sample was composed by different university students belonging to different fields of study and educational level, that we modeled as two relevant covariates. Precisely, we hypothesized an effect of the field of study on the achieved scores in mediating the role of personality traits, possibly depending on the type of majors. Indeed, students enrolled in different majors showed to be characterized by different level of personality traits (see Vedel, 2016, for a review). We also considered the role of educational levels (years of education), postulating that personality and IQ may change their weight on cognitive competences during the students' university career, evidencing how personality and cognition interact along the academic path. Previous works showed the influence of personality on competence assessment scores by comparing groups of participants highly differing in age (Graham and Lachman, 2014; Wettstein et al., 2017), but they did not explore the differences based on the progression along the education. Nevertheless, students' personality traits are likely to change under the influence of the many different emotional and social experiences occurring during their academic career (students make new relationships, strengthen their independency from their family, and acquire new knowledge and habits, see Parker et al., 2004). It is, therefore, plausible that years of education, but also that environment of a specific major, could impact on competences.

Braun and Mishra (2016) highlighted that in higher education the existing approaches for assessing competences are suitable for measuring only one type of skills, that are either cognitive or non-cognitive, and the need to use these approaches in combination. With this study we look forward to underlining the importance of an integrated evaluation of cognitive and non-cognitive skills by testing how a fine-graded cognitive competence assessment test, as the TECO-T, can account for both intelligence and personality. Showing that higher level of cognitive competences would be related not only to higher level of IQ, but also to different level of personal disposition, would be an additional proof of the relevance of using cognitive tests for students' assessments of competences that do not depend only on cognitive skills. It would be also an additional motivation for implementing fine-graded competence assessment tests able to measure a richer set of generic competences, but also to adapt educational policies and strategies in order to plan interventions dedicated to cognitive and non-cognitive skills during university.

## Materials and Methods

### Participants

In the current study we recruited 186 adult participants. Recruitment was pursued through online channels and fliers placed at university sites. It was voluntary-based, and no strict criteria were imposed on their inclusion, except the university enrolment. Before the experimental session, we collected personal information as summarized in Table 1. Years of education correspond to the number of years that each participant spent within the educational system. The sample included students from freshmen and undergraduate to graduate students, with the period of total education ranging between 13 and 21. As to the field of study, the major or degree courses attended by participants were categorized into four main categories: health sciences, scientific sciences, social sciences, and arts and humanities^6^. Overall, there were more females than males in the sample, most of whom attended social sciences and art and humanities. This is in line with the fact that females are usually overrepresented in such fields of study (WEF, 2020).

**Table 1:** Demographic characteristics of the sample.

			**Totals**
Gender	Female	*N*	122
	Male	*N*	64
Age		*Mean (SD)*	22.7 (4.1)
Education (years)		*Mean (SD)*	15.4 (1.6)
Field of study	Health science	*N*	29
	Scientific science	*N*	48
	Social science	*N*	66
	Arts and humanities	*N*	36
Total		*N*	186

*Seven of the total 186 participants did not provide information on the field of study*.

All participants signed in an informed consent prior to the experimental session and received a monetary reimbursement of 10 Euros for their participation. The study was approved by the SISSA^7^'s Ethics Committee.

### Tasks and Procedure

Data collection occurred between 2018 and 2019. Participants individually attended the experimental session in a quiet laboratory, with the supervision of an experimenter. After filling in the questions about personal information (reported in Table 1), they completed the questionnaires and tests described below. The order of the tasks was the same for all participants, except for 43 participants who completed the Big Five Inventory online, 1–4 weeks prior to the experimental session. The tasks, as well as personal questions, were computerized and administered through Google Forms.

The entire experimental session lasted approximately 1 h.

#### Raven's Progressive Matrices

A nine-items scale of the Raven's standard progressive matrices test was used (Bilker et al., 2012). Each participant's scores were calculated according to Bilker et al. (2012), obtaining a prediction of the total score on the 60-items scales that was then converted in a total IQ score according to the age-appropriate standardization procedure (Raven, 1938).

#### Big Five Inventory

The Big Five Inventory (BFI) is a questionnaire consisting in 44 items, eight for Extroversion and nine for each of the remaining traits (Openness, Conscientiousness, Extroversion, Agreeableness, Neuroticism). Participants responded on a 5-point Likert scale from “Strongly disagree” to “Strongly agree” (John et al., 1991; Italian version: Ubbiali et al., 2013, with a Cronbach' Alpha between 0.69 and 0.83). The mean of the scores for each trait was calculated.

#### TEst of COmpetences

Cognitive competences were assessed using the Literacy and Numeracy tests (TEst of COmpetences, TECO-T) developed by ANVUR for the TECO project (Rumiati et al., 2018). The Literacy test is meant to evaluate the undergraduates' levels of understanding and reflect competencies on a text with a generic content. The test contains two types of items: the former requires participants to complete 10 multiple-choice questions after reading a text (text comprehension) and the latter requires them to complete a short text with 20 words that are missing (Cloze test), for a total of 30 items. The Numeracy test assesses undergraduates' levels in logical thinking and solving quantitative problems. This test requires to solve multiple-choice questions: five questions about a short text that includes graphs and tables, five questions about an infographic, and 15 short logical reasoning questions, for a total of 25 items. Reliability (i.e. internal consistency) was derived from the 2016 ANVUR trial^8^ More specifically, the Cronbach's Alphas were, respectively, 0.77 for Literacy and 0.83 for Numeracy. To compute TECO-T's scores two parameter IRT (Item Response Theory) models were used, one for Literacy test one for Numeracy test, in order to test the difficulty and discrimination level for each item of the tests (Rumiati et al., 2018; for a technical description of IRT models see De Boeck and Wilson, 2004). The TECO-T scores are presented as standardized values on a scale with mean 200 and standard deviation 40.

### Data Analysis

To test the relationship between personality and IQ with competences, after testing the non-normal distribution of the continuous variables, we performed Spearman correlation analyses between the mean scores of the Big Five traits, the IQ scores and literacy and numeracy scores. Consistently with our aim to test the relative contribution of intelligence and personality on the acquisition of cognitive competence considering several possible predictors, we conducted a set of regression analyses on the whole sample. Firstly, we separately ran regression models on literacy and numeracy as dependent variables in separate blocks, incrementally adding our predictors of interest. In the first block, we considered the role of the control variables: gender, age, year of education (step A), and field of study (step B). Then, in two further blocks, we explored the predictive power of IQ (steps C and D) and Big Fives (steps E and F) considering them concurrently with the previously included control variables. Lastly, in order to estimate the cumulative power of IQ and Big Fives, we added these dimensions in a unique block, excluding (step G) or including (step H) the field of study.

All analyses and graphic representations were performed using STATA 14 (StataCorp., 2015). In our models we excluded all the cases with missing values on the variables at study. Specifically, we excluded the IQ score of one participant and the attended major of seven participants. In all the tables in the Result session, we reported the sample size that effectively entered each model.

## Results

Table 2 illustrates the descriptive statistics of the dependent and independent continuous variables considered in our analysis, along with the results of the skewness/kurtosis normality test (adjusted chi-squared χ^2^- and *p*-value). Variables were normally distributed with exception of IQ and Openness.

**Table 2:** Descriptive statistics and Skewness/Kurtosis normality test (adjusted chi-squared χ^2^ and *p*-value) of IQ, Big Fives, Literacy, and Numeracy.

	**Mean**	***SD***	**Skewness**	**Kurtosis**	**Adj. **χ**^**2**^**	***p*-Value**
IQ	121.22	9.93	−2.04	7.99	66.67	0.000
Extraversion (E)	3.33	0.79	−0.37	2.68	4.96	0.084
Agreeableness (A)	3.70	0.56	−0.26	2.66	3.14	0.208
Conscientiousness (C)	3.67	0.70	−0.37	2.77	4.70	0.096
Neuroticism (N)	3.03	0.81	−0.14	2.81	0.78	0.678
Openness (O)	4.07	0.60	−1.13	4.93	31.37	0.000
Literacy	200^a^	40^a^	−0.26	2.90	2.26	0.322
Numeracy	200^a^	40^a^	−0.31	2.79	3.22	0.200

a *The TECO scores are presented as standardized values on a scale with mean 200 and standard deviation 40*.

The correlation matrix (Spearman's rho) showed that literacy and numeracy correlated with each other, and they also both correlated with the IQ, while numeracy negatively correlated with Extraversion (see Table 3).

**Table 3:** Correlations (Spearman's rho) between: IQ, Big Fives, Literacy, and Numeracy scores—correlation coefficients, sig. (*p < *0.1*, **p < *0.05*, ***p < *0.01*, ****p < *0.001*).

	**IQ**	**E**	**A**	**C**	**N**	**O**	**Literacy**	**Numeracy**
IQ	1.000							
Extraversion (E)	−0.175**	1.000						
Agreeableness (A)	0.094	0.196***	1.000					
Conscientiousness (C)	−0.058	0.322****	0.208***	1.000				
Neuroticism (N)	−0.025	−0.251***	−0.104	−0.302****	1.000			
Openness (O)	0.010	0.178**	0.027	0.092	0.026	1.000		
Literacy	0.515**	−0.108	0.042	−0.127	0.047	0.047	1.000	
Numeracy	0.512****	−0.173**	−0.016	−0.048	−0.030	−0.091	0.334****	1.000

The regression on the overall sample with the background variables of gender and age did not show a significant effect on literacy and neither did the education expressed in years, which led only to a marginal effect (see also the *R*-squared in Table 4). In contrast, gender and age differences showed a significant positive effect on numeracy, with males obtaining higher scores, while age showed a significantly negative effect. The effect of years of education turned also out to be significantly positive. The addition of the field of study (Table 4, B) seemed to partially absorb the variance explained in the previous model by the years of education for both literacy and numeracy; in contrast, the negative effect of age on numeracy increased while the effect of the field of study showed that participants who attended a major in health or scientific sciences outperformed those in social sciences and art and humanities in the Numeracy test.

**Table 4:** Regression models for Literacy and Numeracy scores by: gender, age, years of education (A), and field of study (B)—coefficients, sig. (*p < *0.1*, **p < *0.05*, ***p < *0.01*, ****p < *0.001*), *N*, and *R*-squared.

		**A**		**B**	
		**Literacy**	**Numeracy**	**Literacy**	**Numeracy**
Gender	Female/Male	1.322	−20.950***	0.987	−17.225***
Age		0.842	−1.648**	0.520	−1.973****
Years of education		3.009*	5.664***	3.199	3.816**
Field of study	Health science/ Art and Humanities			7.859	28.800***
	Scientific science/ Art and Humanities			11.507	34.151****
	Social science/ Art and Humanities			3.620	7.036
Constant		133.644****	163.907****	131.658****	179.957****
*N*		186	186	179	179
*R*-squared		0.0319	0.1009	0.0437	0.2237

Models presented in Table 5 included, in addition to the background characteristics, the IQ which had a positive marginal effect on literacy but a highly significant one on numeracy. IQ together with the field of study (Table 5, D) seemed to account for the variance that in the previous models was explained by the years of education. Interestingly, the strong effect of IQ on numeracy adsorbed that of age which was maintained after introducing the field of study. Furthermore, the field of study confirmed a significant disadvantage in numeracy for those who attended social science and art and humanities studies.

**Table 5:** Regression models for Literacy and Numeracy scores by: gender, age, years of education, IQ (C) and field of study (D)—coefficients, sig. (*p < *0.1*, **p < *0.05*, ***p < *0.01*, ****p < *0.001*), *N*, and *R*-squared.

		**C**		**D**	
		**Literacy**	**Numeracy**	**Literacy**	**Numeracy**
Gender	Female/Male	1.694	−19.789****	0.702	−17.957***
Age		1.052	−1.038*	0.718	−1.443***
Years of education		2.104	3.056*	2.578	2.169
IQ		0.564*	1.660****	0.556*	1.493****
Field of study	Health science/ Art and Humanities			5.488	22.420**
	Scientific science/ Art and Humanities			7.450	23.324***
	Social science/ Art and Humanities			1.998	2.681
Constant		74.191*	−11.962	71.483	18.164
*N*		185	185	178	178
*R*-squared		0.0474	0.2663	0.0586	0.3457

When the predictive effect of the Big Five traits was tested (Table 6), we observed that extraversion had a negative marginal effect on numeracy (0.1 < *p*s > 0.05), that disappeared when the field of study was entered in the model. Gender, age, and years of education remained significant as well as the positive effect of the numeracy scores of those attending scientific and health science majors. Regarding literacy, the addition of the Big Fives turned out to increase the role of years of education which resulted to be significant; when the field of study was included (Table 6, F), conscientiousness marginally increased its negative effect.

**Table 6:** Regression models for Literacy and Numeracy scores by: gender, age, years of education, Big Fives (E), and field of study (F)—coefficients, sig. (*p < *0.1*, **p < *0.05*, ***p < *0.01*, ****p < *0.001*), N, and *R*-squared.

		**E**		**F**	
		**Literacy**	**Numeracy**	**Literacy**	**Numeracy**
Gender	Female/Male	2.639	−19.828***	1.263	−16.084**
Age		0.623	−1.831***	0.276	−2.111****
Years of education		3.611**	6.452****	4.181**	4.640**
Extraversion		−4.029	−7.045*	−3.036	−5.306
Agreeableness		5.580	7.171	8.026	6.890
Conscientiousness		−6.926	−3.259	−8.316*	−4.051
Neuroticism		−0.746	0.919	−1.463	−1.404
Openness		4.970	−4.518	6.047	−6.356
Field of study	Health science/ Art and Humanities			5.874	26.937***
	Scientific science/ Art and Humanities			10.515	34.568****
	Social science/ Art and Humanities			2.372	6.736
Constant		128.700***	185.304****	113.490**	207.277****
*N*		186	186	179	179
*R*-squared		0.0583	0.1372	0.0767	0.2583

When the Big Five traits and the IQ were included in the same models (Table 7), results previously obtained were confirmed. As to numeracy, gender maintained its effect while years of education was absorbed by the IQ as well as age which recovered its negative effect after considering the field of study. As to literacy, considering IQ, Big Fives and field of study together increased the positive effect of years of education, while the slightly negative effect of conscientiousness was maintained.

**Table 7:** Regression models for Literacy and Numeracy scores by: gender, age, years of education, IQ, Big Fives (G), and field of study (H)—coefficients, sig. (*p < *0.1*, **p < *0.05*, ***p < *0.01*, ****p < *0.001*), *N*, and *R*-squared.

		**G**		**H**	
		**Literacy**	**Numeracy**	**Literacy**	**Numeracy**
Gender	Female/Male	2.707	−19.579***	0.819	−17.459***
Age		0.821	−1.189*	0.461	−1.573***
Years of education		2.703	3.516*	3.512*	2.657
IQ		0.495*	1.609****	0.495	1.425****
Extraversion		−3.214	−4.415	−2.382	−3.354
Agreeableness		4.193	2.671	7.028	3.960
Conscientiousness		−6.171	−0.809	−7.706*	−2.266
Neuroticism		0.052	1.657	−0.738	0.752
Openness		4.908	−4.704	6.273	−5.754
Field of study	Health science/ Art and Humanities			4.087	21.855**
	Scientific science/ Art and Humanities			6.982	24.258***
	Social science/ Art and Humanities			0.939	2.615
Constant			12.387	57.754	47.563
*N*		185	185	178	178
*R*-squared		0.0701	0.2848	0.0871	0.3648

### Further Analyses on Participants' Education Level

In this section, we carried out an explanatory *post-hoc* investigation on the effect of the education level. We observed an interesting fluctuation of the effect of years of education which appeared to play a role only when personality was included in the model, but lost its significant impact when IQ was considered. Even if our sample was not a-priori balanced according to years of education, we proposed that further analyses on different subsamples could disentangle the role of personality and IQ along the academic path. Indeed, we further investigated our results by testing whether the effects of the predictors of interest (personality traits and IQ) might depend on when, in the academic career, the students were tested. This was investigated by comparing the same models on three subsamples of participants: freshman (participants with <15 years of education), undergraduates (participants with 15–16 years of education), and bachelor graduates (participants with more than 17 years of education). Given the stability of IQ during adulthood (see e.g., Kautz et al., 2014) and the malleability of personality traits during higher education (Parker et al., 2004), we predicted that students' personality traits, unlike IQ, would change depending on the stage of their academic career.

Despite the limited number of participants in each sub-group, we certified the normal distribution of the parameters (with the exception of IQ and Openness, see Table 8) and proceeded to the following analysis.

**Table 8:** Descriptive statistics and Skewness/Kurtosis normality test (adjusted chi-squared χ2 and *p*-value) of IQ, Big Fives and Literacy and Numeracy by higher educational level.

		**Mean**	***SD***	**Skewness**	**Kurtosis**	**Adj. χ^**2**^**	***p*-value**
Freshmen	IQ	121.19	9.03	−1.417	3.948	16.98	0.000
	Extraversion (E)	3.31	0.81	−0.191	2.525	1.21	0.547
	Agreeableness (A)	3.74	0.53	−0.102	2.124	5.58	0.062
	Conscientiousness (C)	3.68	0.65	−0.109	2.338	2.31	0.315
	Neuroticism (N)	2.93	0.74	−0.923	3.068	0.37	0.832
	Openness (O)	4.08	0.62	−1.447	6.279	23.02	0.000
	Literacy	200^a^	40 ^a^	0.048	2.466	1.02	0.599
	Numeracy	200^a^	40 ^a^	−0.302	2.732	1.41	0.495
Undergraduate	IQ	118.76	12.02	−1.767	6.769	26.93	0.000
	Extraversion (E)	3.37	0.81	0.430	2.715	2.61	0.272
	Agreeableness (A)	3.64	0.57	−0.072	2.603	0.37	0.832
	Conscientiousness (C)	3.58	0.75	−0.476	2.886	3.10	0.212
	Neuroticism (N)	3.06	0.88	−0.211	2.642	0.83	0.660
	Openness (O)	4.04	0.63	−0.838	3.493	8.25	0.016
	Literacy	200^a^	40 ^a^	−0.585	3.451	5.37	0.068
	Numeracy	200^a^	40 ^a^	−0.350	2.944	1.79	0.410
Bachelor graduate	IQ	126.22	2.54	−2.780	10.658	29.50	0.000
	Extraversion (E)	3.31	0.71	−0.733	2.970	4.20	0.122
	Agreeableness (A)	3.74	0.59	−0.799	3.538	5.48	0.065
	Conscientiousness (C)	3.83	0.65	−0.482	2.719	1.92	0.383
	Neuroticism (N)	3.16	0.80	−0.196	2.703	0.32	0.854
	Openness (O)	4.14	0.49	−0.658	3.260	4.06	0.131
	Literacy	200^a^	40 ^a^	0.129	2.869	0.21	0.901
	Numeracy	200^a^	40 ^a^	−0.241	2.419	0.89	0.640

a *The TECO scores are presented as standardized values on a scale with mean 200 and standard deviation 40*.

**Figure 1:**
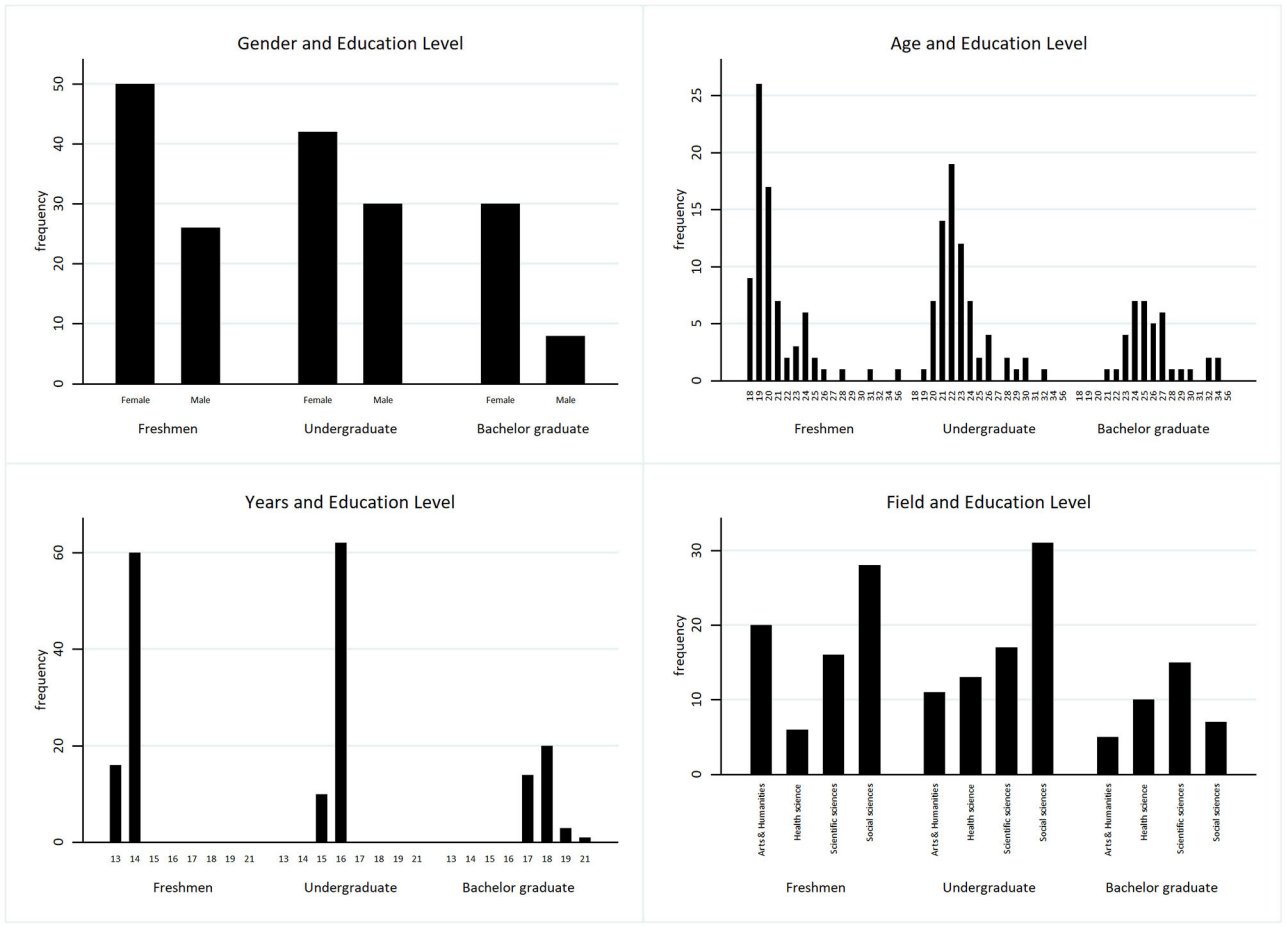
Distribution of gender, age, years of education and field of study according to different educational levels (freshmen, undergraduates, and bachelor graduates).

After testing the Spearman's correlation between Big Five traits, IQ and the achieved tests scores, separate regression models were performed for each sub-groups of participants exploring the power of the predictors of interests (step I) by adding the field of study (step J) on literacy and numeracy scores. Considering the background variables in relation to the different levels in higher education, differences in age were observed, but also differences in the feminization of the participants and their fields of study (Figure 1).

Differences depending on the educational level were observed in the correlations between Big Five and cognitive skills (Table 9). As observed for the whole sample, literacy and numeracy variously correlated with each other and with the IQ. Considering the correlation between competence scores and personality, while only numeracy and extraversion significantly correlated in the whole sample, numeracy maintained a mildly negative correlation with extraversion only for freshmen, with an addition of a mildly negative correlation with consciousness and openness for the undergraduates. Additionally, a significant and negative correlation was revealed between literacy and conscientiousness only for undergraduates.

**Table 9:** Spearman's rho by higher education level between: IQ, Big Fives, Literacy, and Numeracy scores—correlation coefficients, sig. (*p < *0.1*, **p < *0.05*, ***p < *0.01*, ****p < *0.001*).

		**IQ**	**E**	**A**	**C**	**N**	**O**	**Literacy**	**Numeracy**
Freshmen	IQ	1.000							
	Extraversion	−0.221*	1.000						
	Agreeableness	0.141	0.278**	1.000					
	Conscientiousness	−0.119	0.328***	0.089	1.000				
	Neuroticism	−0.210*	−0.349***	−0.084	−0.332***	1.000			
	Openness	0.143	0.030	0.168	−0.055	−0.107	1.000		
	Literacy	0.132	−0.090	0.065	−0.121	0.131	0.090	1.000	
	Numeracy	0.498****	−0.184	−0.113	−0.036	−0.075	0.034	0.282**	1.000
Undergraduate	IQ	1.000							
	Extraversion	−0.162	1.000						
	Agreeableness	0.066	0.052	1.000					
	Conscientiousness	−0.050	0.336***	0.199*	1.000				
	Neuroticism	0.014	−0.168	−0.105	−0.386****	1.000			
	Openness	−0.151	0.345***	−0.276**	0.157	0.144	1.000		
	Literacy	0.147	−0.220*	−0.011	−0.272**	0.034	−0.090	1.000	
	Numeracy	0.504****	−0.161	0.102	−0.153	−0.140	−0.269**	0.489****	1.000
Bachelor graduate	IQ	1.000							
	Extraversion	−0.125	1.000						
	Agreeableness	0.031	0.284*	1.000					
	Conscientiousness	−0.106	0.319*	0.412**	1.000				
	Neuroticism	0.131	−0.253	−0.016	−0.129	1.000			
	Openness	−0.085	0.192	0.302*	0.263	−0.012	1.000		
	Literacy	0.179	0.089	0.129	0.196	−0.239	0.230	1.000	
	Numeracy	0.485***	−0.214	0.075	0.029	0.158	−0.058	0.176	10.000

*^a^The TECO scores are presented as standardized values on a scale with mean 200 and standard deviation 40*.

Table 10 summarizes the results of the regression models performed for each subsample in which background characteristics, IQ, and personality traits were included as predictors. Age turned out to be a relevant factor within the freshman group, mildly positive for literacy and significantly negative for numeracy. Gender showed a strong effect on numeracy for freshmen and bachelor graduates. The IQ confirmed a significant positive relation with numeracy across the education levels, with also male freshmen and bachelor graduates presenting significant better scores on numeracy. The IQ effect on literacy appeared only marginal for freshmen and bachelor graduates. Regarding undergraduates' personality traits, conscientiousness showed a marginal negative relation with literacy (observed also in the whole sample), while it strongly correlated with numeracy, but with an opposite sign, in bachelor graduate sample.

**Table 10:** Regression models by higher education level (I1, I2, I3) for Literacy and Numeracy scores by: gender, age, IQ, Big Fives—coefficients, sig. (*p < *0.1*, **p < *0.05*, ***p < *0.01*, ****p < *0.001*), *N*, and *R*-squared.

		**Freshmen**		**Undergraduates**		**Bachelor graduates**	
		**(I1)**		**(I2)**		**(I3)**	
		**Literacy**	**Numeracy**	**Literacy**	**Numeracy**	**Literacy**	**Numeracy**
Gender	Female/Male	−1.796	−31.279****	10.399	−5.646	−3.138	−32.934***
Age		0.753*	−1.768****	1.418	0.699	1.338	0.128
IQ		1.157*	1.938****	0.305	1.192**	2.390*	9.263****
Extraversion		4.171	0.348	−6.553	−1.949	−0.354	−13.313*
Agreeableness		2.546	−6.809	−2.741	3.909	2.067	4.121
Conscientiousness		−0.237	4.791	−12.712*	−12.284	7.159	13.431**
Neuroticism		11.049	5.867	−4.160	−6.991	−7.857	9.447
Openness		7.673	−3.989	−0.131	−5.791	13.637	−7.653
Constant		−49.011	24.744	219.573***	125.116	−189.640	−956.855***
*N*		76	76	72	72	37	37
*R*-squared		0.0948	0.4165	0.1043	0.2488	0.179	0.5063

As apparent in the models reported in Table 11, the field of study confirmed its influence only for freshmen, with an advantage on numeracy scores for those who within health and scientific sciences. Furthermore, the field of study did not alter the IQ influence on numeracy, except for an attenuated effect in undergraduates. Interestingly, the IQ showed a significant correlation on literacy in freshmen. When the Big Fives were introduced, literacy showed a marginal positive relation with openness among the freshmen, and a marginal negative relation with neuroticism in bachelor graduates, while conscienceless turned to be significantly negatively related with numeracy among undergraduates.

**Table 11:** Regression models by higher education level (J1, J2, J3) for Literacy and Numeracy scores by: gender, age, IQ, Big Fives, and field of study—coefficients, sig. (*p < *0.1*, **p < *0.05*, ***p < *0.01*, ****p < *0.001*), *N*, and *R*-squared.

		**Freshmen**		**Undergraduates**		**Bachelor graduates**	
		**(J1)**		**(J2)**		**(J3)**	
		**Literacy**	**Numeracy**	**Literacy**	**Numeracy**	**Literacy**	**Numeracy**
Gender	Female / Male	-5.487	−24.572***	10.029	−5.583	0.618	−32.404**
Age		0.657	−2.108****	1.019	−0.406	0.923	−0.168
IQ		1.501**	1.810***	0.330	1.072*	1.172	7.925***
Extraversion		6.003	3.445	−5.819	−0.401	−0.323	−11.963
Agreeableness		8.558	−8.523	−0.992	6.367	8.091	8.739
Conscientiousness		−0.481	3.204	−14.695*	−15.333**		11.134
Neuroticism		10.404	1.766	−4.348	−6.331	−10.379*	8.735
Openness		12.945*	−7.660	0.157	−7.169	19.239	−0.978
Field of study	Health science/ Art and Humanities	−0.227	33.614**	−14.185	2.070	14.694	19.482
	Scientific science/ Art and Humanities	10.027	30.575***	−5.790	12.444	31.988*	26.464
	Social science/ Art and Humanities	13.289	−8.607	−19.063	−14.210	14.111	14.508
Constant		−142.001	56.669	35.695***	167.754	−74.541	−837.313***
*N*		70	70	72	72	36	36
*R*-squared		0.1658	0.5641	0.1353	0.3191	0.2943	0.5868

## Discussion

As to the main aim of the study, we generally confirmed our predictions that both IQ and personality affect cognitive competences, also when years of education and fields of study are controlled. More specifically, when both IQ and personality traits were included as predictors, the model fit increased, indicating an overall weight of both personality and cognition on the cognitive assessment scores. Interestingly, our results provide an important insight into their differential effects on literacy and numeracy scores. Regarding the IQ, we confirmed previous findings on its effect on cognitive abilities (e.g., Borghans et al., 2016). However, we found it highly significant in predicting numeracy scores but marginally significant in predicting literacy scores. This pattern was observed when entering only the IQ in the models (C and D) where it explained around 12–16% of the variance on numeracy scores, and 1–2% on literacy scores. When only personality was considered (models E and F), the five personality traits accounted for around 4% of the variance on literacy scores and around 1–2% on numeracy scores (whether the field of study was or was not considered). In sum, compared to IQ, personality seems to have a more prominent effect on literacy while IQ maintained a highly significant relation with numeracy. This appears partially at variance with previous findings. In detail, in the extant literature, the impact of both IQ and personality on cognitive abilities has been analyzed using a wide variety of constructs and tests for the latter, and generally considering overall competence scores rather than specific competence (such as literacy or numeracy) scores (e.g., Duckworth et al., 2012; Borghans et al., 2016). This is in line with the enhanced variances we observed in both cognitive tests. However, when the effect of personality was analyzed separately for literacy and numeracy (based on PIAAC exercise of 2012), Rammstedt et al. (2016, 2017) found the same significant associations and almost the same pattern of results in the interaction between personality traits and educational level, as well as between personality and labor force participation. Even though the authors did not consider the concomitant effect of IQ, their findings are discordant with what we found here as personality exerts a moderate effect on either competence, slightly stronger for literacy.

Differences in specific personality traits did not turn out significant in either literacy or numeracy, even if we expected to observe positive correlations with openness and negative correlations with neuroticism and conscientiousness. The unpredicted significant negative correlation between extraversion and numeracy scores was reflected in a mild tendency in the regression model E, which considers the Big Fives without the field of study as control predictors. This relation has been reported before in the literature even though not consistently. Indeed, a negative relation between extraversion and both crystalized and fluid intelligence was previously reported and explained as a greater prominence of less extraverted people in intellectual efforts than in social relations (e.g., Soubelet and Salthouse, 2011; Malykh, 2017). Another mild although not significant negative relation was observed between conscientiousness and literacy, which resisted across models, also when IQ was considered. This negative relation is consistent with previous research findings (e.g., Moutafi et al., 2003, 2004, 2006; Furnham et al., 2005, 2007; Wood and Englert, 2009; Soubelet and Salthouse, 2011; Furnham and Moutafi, 2012; Rammstedt et al., 2016, 2017). It is usually interpreted within the intelligence compensation hypothesis (ICH) according to which people with lower level of intelligence compensate their difficulties by becoming more conscientious in order to emulate their peers; in contrast, those with higher level of intelligence do not need to compensate (Moutafi et al., 2004). However, Murray et al. (2014) suggested that this negative relation might be due to compensatory selection of the samples, that usually included individuals in competitive professional or educational settings that require a certain level of achievement. Our sample was composed of college students at different stages of the university career. Since admission to an Italian public university does not generally require an high score on specific competence tests, the negative effect observed for conscientiousness could be due to undergraduates' and bachelor graduates' self-selection. The analysis of the different subsamples' results discussed below provides some support to this latter view.

As to the covariates, as we have hypothesized, in the overall sample, significant and different roles on cognitive competences were reached by years of education and field of studies, but also by age and gender. In particular, age decreased with numeracy ability while years of education increased, and males outperformed female participants. The males' advantage in numeracy has recently been reported to be smallest at the age of 10 and largest at 27, picking from 15 (Borgonovi et al., 2018). Selective numeracy effects were observed also when considering the field of study, with participants attending scientific and health sciences outperforming those in social sciences and art and humanities. The latter effect could be related to the decrement of numerical skills training in higher education, which seems to depend on whether numeracy is actively practiced in the study program as it happens in the scientific sciences (for similar results, see also Rumiati et al., 2018). Years of education turned out to be unstable, and plaid a role only when personality was included in the models, but lost its significant impact when IQ was considered. We proposed that personality might exert differential effects depending on the progression of university career.

Based on this latter idea, we further analyzed the impact of cognitive and non-cognitive factors on three sub-groups of participants: freshmen, undergraduates, and bachelor graduates. Although individual parameters were equally distributed across groups, we are aware that the size of overall sample was not large, and therefore we interpreted the results as being only exploratory.

The of freshmen, influence of IQ and personality on cognitive competences undergraduates and bachelor graduates led to different results depending on the students' university career level. First, IQ was found to correlate more strongly with numeracy than with literacy. However, after entering the field of study in the model, a significant positive association with freshmen's literacy scores emerged while no significant relation was found for undergraduates. How can we explain these differential effects depending on the students' level of their university career? The positive association between IQ and both literacy and numeracy showed by freshmen suggests that intelligence plays a role in predicting both competence scores; indeed, for these students both the abilities acquired at school, are still active, and in Italian high schools they are taught independently of the school type. For students who are more advanced in their career (as undergraduates)—and depending on the field of study—, the IQ loses therefore its predictive power, and personal dispositions become more influential. We know also that intelligence showed to be rather stable with age (e.g., Kautz et al., 2014), while personality is more malleable (i.e., Poropat, 2009; Graham and Lachman, 2014; Wettstein et al., 2017) and differs depending on the major attended (Vedel, 2016). In line with this reasoning, undergraduates showed a pronounced negative effect of conscientiousness on numeracy and a trend on literacy. Consequently, in the overall sample analysis, this negative association occurs mainly due to undergraduates, in line with the ICH (Moutafi et al., 2004) or with the compensatory selection (Murray et al., 2014). Conscientiousness, on the other hand, did not affect neither freshmen' nor bachelor graduates' scores except for a positive association with numeracy for bachelor graduates when the field of study was not considered. Both non-significant correlations (e.g., Chamorro-Premuzic et al., 2005; Furnham et al., 2005; Bartels et al., 2012) and positive correlation (e.g., Lounsbury et al., 2005; Baker and Bichsel, 2006; Luciano et al., 2006; Malykh, 2017) between contentiousness and cognition were previously found. Interestingly, this latter result is consistent with studies reporting a correlation between conscientiousness and academic success (Busato et al., 2000; Poropat, 2009), suggesting that such trait could be a good predictor of cognitive competences in academically more advanced students besides intelligence. Nevertheless, the field of study absorbed this association, probably due to the differential distribution of the personality traits or other unobserved characteristics which could not be controlled for, due to the limited size of this sub-sample.

In addition, two further results observed in the models are worth to be discussed despite they were not significant, as their trend is in line with previous findings. Openness had a mildly positive association with literacy in freshmen, and neuroticism had a negative association with literacy in bachelor graduates. Although we expected a generally strong relation between intelligence and openness across all subgroups (Soubelet and Salthouse, 2011; Kretzschmar et al., 2018), this mildly positive association was observed only in students at the beginning of their university career. This result is in line with a previously observed positive correlation between openness and academic performance, declining with the secondary and tertiary level of education as well with students' age (Poropat, 2009). Regarding neuroticism, stable findings about the negative correlation with intelligence was found in the literature across different ages (e.g., Ackerman and Heggestad, 1997; Moutafi et al., 2003). Our finding shows a slightly negative impact on literacy in bachelor graduates that is in line with a correlation between emotional stability (the counterpart of neuroticism) and intelligence that decreases as age increases (Poropat, 2009). Indeed, our sample of bachelor graduates presented an average increased age compared with the other sub-samples. Furthermore, consistently with Chamorro-Premuzic et al. (2006), neuroticism impaired verbal skills (literacy) but not numerical ones (numeracy).

## Conclusions

The main aim of the study was to quantify the relative influence of intelligence and personality traits on literacy and numeracy scores of students enrolled in higher education, controlling for the effect of field of study and years of education. Furthermore, we explored the contribution of personality and intelligence on competences at different stages of the university career (freshmen, undergraduates, and bachelor graduates). Our predictions were partially confirmed and integrated by the unexpected differences between literacy and numeracy results. IQ generally had a high impact on numeracy skills and a moderate or no impact on literacy skills, while personality seemed to affect literacy more than IQ did. Interestingly, the link between personality, intelligence, and outcomes on competence tests seemed to change depending on the students' university career level, particularly in the case of conscientiousness. Only undergraduates showed a negative correlation between conscientiousness and tests scores, and post-graduates showed a positive correlation: to advance in their academic career students require higher competence levels, and to compensate lower competences levels higher level of conscientiousness kicks in. On the other hand, after graduation, both characteristics have an effect on achieved scores. This is consistent with the lack of an IQ significant effect on both literacy and numeracy in undergraduates, that might have been absorbed by the personality trait. Furthermore, high levels of openness partially predicted literacy achieved scores only in freshmen and low levels of neuroticism in bachelor graduates. This suggests that different personal dispositions increase or decrease their impact on competencies depending on the educational level. Finally, the field of study turned out to be a predictor of numeracy, but also an important covariate in the models altering the pattern of personality impact.

This study has some limitations. The main one is the sample size which makes some results difficult to interpret and prevents us from deriving strong conclusions, especially with respect to the different subsamples. Nevertheless, our results make more plausible future research on the impact of personality on cognitive competences depending on the university career. Furthermore, we used competence tests that have never been tested before in combination with personality and IQ, thus making it difficult to compare our results with other studies.

Even though our sample is not large, our results support the importance of comparing different levels of higher education, in addition to what is already known about comparing individuals with larger age differences. For this reason, we encourage future research to deeply explore the contribution of personality traits and non-cognitive skills on cognition in extended sample size and through stratified sample selection not only depending on the level of education but also on the field of study. Additionally, our research suggests that literacy and numeracy should be studied as two separate competences. Further research should be directed also to evaluate the role of non-cognitive skills on literacy and numeracy separately, beyond composite scores. Indeed, cognitive competences do not develop simultaneously and could be differently affected by personal disposition. Regarding personality, a lot remains to be done in reference to specific traits along education, as to understand whether they have a role in predicting other generic competences (instrumental, interpersonal, and systemic), and how these competences are in turn interrelated.

To conclude, understanding how and when personality interact with cognition and cognitive competences along higher education can help in evaluating existing educational strategies and in planning *ad hoc* interventions, not only in view of academic success, but also to build up the competences for the future of the labor market (see e.g., Halász and Michel, 2011; Graczyk-Kucharska et al., 2018). Future effort should be directed to understand how personality and cognition prepare students not only to cope with competence assessments but also to transfer their competences beyond the university. We refer to the lifelong learning outcomes such as personal fulfillment, a healthy and sustainable lifestyle, employability, active citizenship, and social inclusion (European commission, 2006, 2018). These interventions should consider not only the original level of cognitive abilities, but also non-cognitive dispositions and the university career (the reached level and the field of study). Given that personality and cognition predict life and career outcomes (Farsides and Woodfield, 2003; Furnham et al., 2005, 2007; Graham and Lachman, 2012) as well as cognitive assessments do (Cappellari et al., 2017), potentiating the first should reinforce the latter, as they beneficially affect personal outcomes.

## Data Availability Statement

The raw data supporting the conclusions of this article will be made available by the authors, without undue reservation.

## Ethics Statement

The studies involving human participants were reviewed and approved by Ethics Committee, Scuola Internazionale Superiore di Studi Avanzati (SISSA). The participants provided their written informed consent to participate in this study.

## Author Contributions

TC and RR contributed to the design of the study. TC was responsible for data collection. AD performed statistical analyses. All authors contributed to results interpretation, manuscript preparation, and agree to be accountable for the content of the work.

## Conflict of Interest

The authors declare that the research was conducted in the absence of any commercial or financial relationships that could be construed as a potential conflict of interest.

## Footnotes

^1^We refer in particular to the *Definition and Selection of Competencies* project *(DeSeCo)* and the following *The Future of Education and Skills 2030* developed by the OECD, and the *Recommendation on Key Competences for Lifelong Learning*, developed by the European Union since 2006 (European commission, 2006, 2018).

^2^Official website: https://www.oecd.org/pisa/

^3^Official website: https://www.oecd.org/skills/piaac/

^4^Agenzia Nazionale per la Valutazione del sistema Universitario e della Ricerca—ANVUR.

^5^The TECO project has a broader extent: other tests have been developed and administered to evaluate problem solving and civics, and the results are being processed to date (Rumiati et al., 2019). Furthermore, ANVUR has supervised the development of disciplinary tests some of which are already been validated and currently used including the health professions (Ciolfi and Di Benedetto, 2019; Crescenzo et al., 2019; Galeoto et al., 2019), pedagogy, philosophy, and medicine (see Bracci and Romano, 2018) while others are in the process of being validated or constructed.

^6^The MIUR (Italian Minister of University and Research) acknowledges three main disciplinary areas (health sciences, scientific sciences, social sciences-humanities). However, we further divided the latter into two distinct areas of social sciences and arts and humanities.

^7^Scuola Internazionale Superiore di Studi Avanzati, the third author's institution. It is a special scientific institute set up with the purpose of preparing graduates for advanced research and focused on three main areas: Physics, Neuroscience, and Mathematics.

^8^https://www.anvur.it/wp-content/uploads/2020/11/LarilevazioneTECO2016.pdf
